# Neural dynamics of audiovisual speech integration under variable listening conditions: an individual participant analysis

**DOI:** 10.3389/fpsyg.2013.00615

**Published:** 2013-09-10

**Authors:** Nicholas Altieri, Michael J. Wenger

**Affiliations:** ^1^Department of Communication Sciences and Disorders, Idaho State UniversityPocatello, ID, USA; ^2^Department of Psychology, The University of OklahomaNorman, OK, USA

**Keywords:** capacity, integration, multisensory speech, models of integration, Late ERPs, audiovisual integration, audiovisual interactions

## Abstract

Speech perception engages both auditory and visual modalities. Limitations of traditional accuracy-only approaches in the investigation of audiovisual speech perception have motivated the use of new methodologies. In an audiovisual speech identification task, we utilized *capacity* (Townsend and Nozawa, 1995), a dynamic measure of efficiency, to quantify audiovisual integration. Capacity was used to compare RT distributions from audiovisual trials to RT distributions from auditory-only and visual-only trials across three listening conditions: clear auditory signal, S/N ratio of −12 dB, and S/N ratio of −18 dB. The purpose was to obtain EEG recordings in conjunction with capacity to investigate how a late ERP co-varies with integration efficiency. Results showed efficient audiovisual integration for low auditory S/N ratios, but inefficient audiovisual integration when the auditory signal was clear. The ERP analyses showed evidence for greater audiovisual amplitude compared to the unisensory signals for lower auditory S/N ratios (higher capacity/efficiency) compared to the high S/N ratio (low capacity/inefficient integration). The data are consistent with an interactive framework of integration, where auditory recognition is influenced by speech-reading as a function of signal clarity.

Studies of audiovisual speech recognition have revealed the dramatic effect that visual information can have on the processing of auditory speech inputs. One of the most significant findings is that visual speech signals provided by a talker's face enhance identification accuracy, especially when listening conditions become degraded (e.g., Sumby and Pollack, 1954; see Ross et al., 2007). Accuracy data from audiovisual speech identification experiments have pointed to a specific range of auditory signal-to-noise (S/N) ratios in which audiovisual integration occurs most efficiently (Ross et al., 2007). For example, Grant et al. (1998) fit models of consonant identification that allow the relative contribution of each information source to be estimated from the data (see Braida, 1991; Massaro, 2004). The authors applied these models to data sets obtained from normal-hearing and hearing-impaired subjects in identification experiments. These studies indicate considerable individual variability in the ability to combine auditory and visual information. This variability has been observed in both normal-hearing and hearing impaired listeners (see Grant et al., 1998).

The implication of these studies is that the visual signal affords variable levels of integration efficiency under different listening conditions. Specifically, this suggests that integration occurs in fundamentally distinct ways under different auditory S/N ratios and across different populations such as normal-hearing vs. hearing-impaired (e.g., Sommers et al., 2005). Also, an important aspect of speech recognition for both unisensory and multisensory cases concerns the temporal nature of the speech signal. Speech recognition unfolds in real-time, and audiovisual speech studies that do not employ measures of the dynamics of processing can miss important characteristics of neural and cognitive mechanisms (Altieri et al., 2011). A unified approach for investigating audiovisual speech integration must combine real-time behavioral measures with dynamic brain signals (Besle et al., 2004; van Wassenhove et al., 2005, 2007; Pilling, 2009; Cappe et al., 2010). This will involve combining EEG amplitude with model based reaction time (RT) methods (see e.g., Altieri, 2010; Altieri and Townsend, 2011; see also Colonius and Diederich, 2010).

Our study utilizes a combined EEG and RT model-based approach to investigate the following questions: (1) under which listening conditions does visual speech information yield the most efficient integration? (2) At which points in time during speech recognition does the visual signal have the greatest influence on the auditory speech percept? And (3), to what extent are neural measures of efficiency predictive of model based behavioral measures of efficiency? This latter point is especially important because EEG amplitude can indicate neural firing associated with sensory processing, extraction of features, and recognition/categorization. For example, one study using a spoken word recognition test in children with hearing loss observed ERPs of approximately normal amplitude and latency in children with better speech recognition, but significantly reduced or absent ERPs in those poor word recognition ability (Rance et al., 2002). Nonetheless, ERP studies have almost universally failed to relate ERPs to a quantitative behavioral index of processing ability that makes predictions relative to a well-defined behavioral model (although cf. Winneke and Phillips, 2011).

To address this latter issue, we obtained a behavioral measure of integration efficiency known as *capacity* that uses non-parametric predictions of parallel independent processing models (Townsend and Nozawa, 1995; Altieri and Townsend, 2011) as a benchmark for efficient integration. While we measure capacity/efficiency behaviorally, “capacity” does not directly refer to processing architecture (e.g., parallel vs. coactive; Miller, 1982; Townsend and Nozawa, 1995). Second, we obtained brain recordings to examine the extent to which ERPs systematically covary with capacity across listening conditions.

## Neuro-cognitive basis of integration efficiency

Evidence obtained from EEG (e.g., Ponton et al., 2009; Naue et al., 2011; Winneke and Phillips, 2011) as well as RT studies (e.g., Altieri and Townsend, 2011; Altieri et al., 2011) is consistent with the hypothesis that unisensory processing occurs in separate channels, with cross-modal interactions occurring between them (cf. Rosenblum, 2005). In the ERP literature, Winneke and Phillips (2011) used a combination of RTs and ERPs to assess integration skills across age group. The analysis of the RT data and pre-linguistic ERP components associated with auditory-visual detection, revealed early dependencies between sensory modalities. The analysis of the N1/P1 components showed an amplitude reduction in both components on audiovisual trials relative the auditory-only plus visual-only trials. However, the precise physiological relationships between patterns of brain signals and variations in integration efficiency, and the manner in which those co-variations relate to the predictions for cross-modal dependencies have yet to be established.

**Figure 1:**
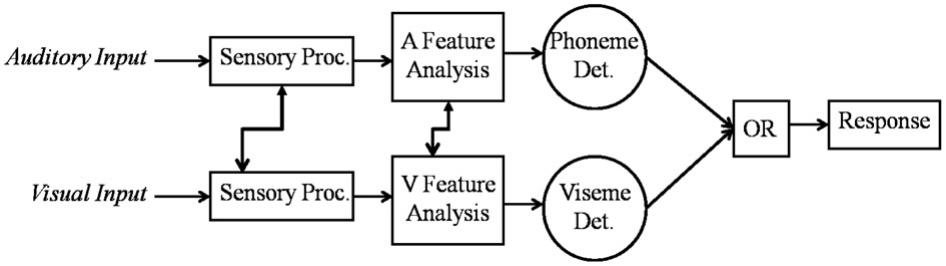
**A parallel model of audiovisual recognition with separate detectors for the auditory and visual channels.** The model allows for cross-modal interaction in the early and later stages of recognition, although our study primarily focuses on obtaining evidence for later interactions.

Moreover, Altieri and Townsend (2011) fit processing models to RT distributions obtained from audiovisual identification data and found that a parallel model, with separate auditory and visual channels and a first terminating (OR) decision rule (see Townsend and Nozawa, 1995; Townsend and Wenger, 2004) best accounted for the data. Figure 1 shows a schema of this model. First, auditory, and visual speech cues are input, which undergo early sensory processing (prior to conscious language recognition). Subsequently, language based features such as phonemes and visual cues about place of articulation (Summerfield, 1987) are extracted, and information related to a percept is accumulated until a decision bound is reached using an OR decision rule. That is, recognition occurs as soon as either enough auditory or enough visual speech information is available (Altieri and Townsend, 2011). To use an example, suppose that as soon as enough auditory evidence for a word/category, say [Date], reaches threshold, the listener perceives “Date.” A critical feature of this model is that we hypothesize that the channels are not independent—hence the arrows showing cross-modal connections. We primarily concern ourselves with audiovisual interactions occurring during linguistic analysis, although evidence exists for earlier interactions. The capacity results will be critical in falsifying null-hypothesis assumptions of independence, pointing instead to dependencies during phoneme/word perception in audiovisual integration.

Although later ERPs occurring post 400 ms have not been commonly analyzed in audiovisual word recognition, they are believed to be associated with phonemic recognition and semantic processing. In one a spoken word recognition study examining the relationship between semantic activation and ERP amplitude, Woodward et al. (1990) uncovered evidence for a large negative peak occurring around 480 ms, followed by a large positive potential peaking approximately 800 ms. The scalp tomography consisted of several frontal and parietal electrodes, and variability in latency and amplitude was believed to correspond to recognition points. Other studies on audiovisual integration have investigated late ERP components associated with conscious language recognition, although this is usually done within the context paradigms in which an “odd-ball” or incongruent signals are detected (cf. Klucharev et al., 2003; Riadh et al., 2004). Later potentials have also been shown to be influenced by the integration of incongruent audiovisual signals (e.g., Riadh et al., 2004; Arnal et al., 2011), and also important for processing phonological information (e.g., Klucharev et al., 2003; Henkin et al., 2009). The importance of analyzing later EEG activation cannot be overstated. In general, later ERP activation will be associated with accessing lexical memory, categorization, semantic processing, and even executive function. All of these functions are vital for language processing—especially under difficult conditions.

We therefore aim to investigate the relationship between audiovisual recognition and integration efficiency in greater detail. This study will establish a systematic relationship between capacity (a mathematical index of integration), and a late ERP related to language processing. We will examine audiovisual integration under easy listening conditions where the visual signal may be of little use, and under degraded listening conditions, where the visual information becomes increasingly helpful. The earlier N1 component will be examined as well.

### Measuring integration efficiency

Integration efficiency can be measured by using a measure of *capacity* [*C(t)*]*—*a cumulative measure of work competed or energy expenditure (Townsend and Nozawa, 1995; Townsend and Wenger, 2004). It is a probabilistically defined RT measure in which independent first-terminating processing establishes a benchmark. Capacity is a measure that compares RTs from trials where auditory and visual information are present, to RTs obtained from trials where either auditory-only or visual-only information is present. The capacity coefficient uses the entire distribution of RTs, at the level of the integrated hazard function. The integrated hazard function is defined as

(1) $$H\left(t^{*}\right)=\int_{0}^{t^{*}}h\left(t\right)dt,\text{ where }h\left(t\right)=\frac{f\left(t\right)}{S\left(t\right)}$$

and *f(t)* is the probability density function and *S(t)* is the survivor function, such that *h(t)* gives the probability of a response in the next instant of time given that the response has not yet occurred (Townsend and Ashby, 1978, 1983; Townsend and Wenger, 2004). The hazard function approach has both conceptual and statistical advantages (see Wenger and Gibson, 2004 for discussion). Crucially, for our integration study, it captures the notion of capacity and “efficient energy expenditure” more closely than mean accuracy or RTs.

The use of capacity can thus be advantageous over mean RTs. First, as we shall see, capacity assays efficiency relative to independent (race) model predictions (Miller, 1982). Independent race models predict that auditory and visual information does not influence each other during processing; however, audiovisual processing is faster than either auditory or visual alone due to purely statistical reasons. Furthermore, context independence refers to the assumption that auditory completion times, for example, are unaffected by whether or not visual information is present (e.g., Townsend and Nozawa, 1995). Deviations from model predictions suggest that the predictions of the independent channels model have been falsified due to either limitations in efficiency or processing resources, facilitatory or inhibitory cross-channel interactions (e.g., Eidels et al., 2011), or perhaps coactivation where the auditory and visual information are pooled into a common processor (Miller, 1982; Townsend and Nozawa, 1995), in which case capacity is much greater than “1.” A second advantage of capacity is that it makes use of integrated hazard functions. Given that the hazard function can be interpreted in terms of the instantaneous intensity of work, the integrated hazard function can be interpreted in terms of the total amount of work completed up until time *t*. Townsend and Nozawa (1995) derived the benchmark capacity coefficient for tasks in which observers are presented with 0, 1, or 2 target stimuli and have to respond if either 1 or 2 stimuli are present. For present purposes, if we let *H*_*AV*_(*t*) denote the integrated hazard function obtained from audiovisual trials, and let *H*_*A*_(*t*) and *H*_*V*_(*t*) denote the integrated hazard functions obtained from the auditory-only and visual-only trials, respectively, then the capacity coefficient is defined as:

(1) $$C(t)=\frac{H_{AV}(t)}{H_{A}(t)+H_{V}(t)}$$

Note that the term in the denominator corresponds to the predictions of an independent race model (Miller, 1982). The capacity coefficient provides a useful non-parametric measure of integration efficiency in a variety of settings, with there being three possible outcomes and associated interpretations. First, the capacity coefficient can be greater than 1 at time *t*, indicating faster RT and thus more work completed in the audiovisual condition compared to the auditory- and visual-only conditions. In this case, we have highly efficient integration since RTs in the audiovisual condition are faster than would be predicted by independent race models. Second, capacity can be less than 1 at time *t*, indicating slower reaction times in the audiovisual condition compared to the unisensory conditions, and therefore inefficient audiovisual integration. A third possibility is that the capacity coefficient can be equal to 1 at time *t*. This would suggest that audiovisual processing is neither faster nor slower and is thus just as efficient as unisensory processing.

## Hypotheses and predictions

We aim to model integration efficiency (i.e., Altieri, 2010; Altieri and Townsend, 2011) at different auditory S/N ratios in an audiovisual word identification task. We will relate neural measures of integration efficiency with behavioral measures of efficiency across variable S/N ratios. To accomplish this, we obtained EEG recordings and compared how peak and time-to-peak amplitudes in the audiovisual condition differed from the uni-sensory conditions as a function of auditory S/N ratio. For comparison purposes, we also report traditional accuracy based measures of integration (“Gain,” e.g., Sumby and Pollack, 1954), although we would argue that accuracy alone does not reflect integration efficiency as meaningfully as capacity.

## Hypotheses

### ERPs

We aim to examine the hypothesis that the visual signal is used more (or less) efficiently as listening conditions change. Furthermore, the neural signal should co-vary with capacity observed in individual participants.

#### Null hypothesis

The null hypothesis for ERP data predicts that the relation between AV ERPs and A-only peak ERPs will remain constant across listening conditions. Of course, the amplitude of the signals should differ as a function of noise (likely decreasing in noisy listening conditions); however, the relative amplitude between AV and A should remain relatively constant. This should be true for the later ERP, and earlier N1 potentials.

#### Alternative hypothesis

We predict that the AV peak amplitude for the late ERP will increase relative the A-only (and possibly V-only) as listening conditions became increasingly degraded. This should mirror changes in capacity (discussed next). First, (1) in the high S/N ratio condition, the peak ERP occurring post 400 ms will be approximately equivalent in the multisensory and unisensory conditions; (2) in the −12 and −18 S/N ratio conditions, the amplitude will be greater in the AV compared to the unisensory conditions. This is because the AV ERP should increase as visual information increasingly assists auditory identification, the latter of which becomes degraded and requires visual place cues to facilitate recognition (Grant et al., 1998). Hence, as A-only accuracy, speed, and amplitude decrease, AV speed, accuracy and therefore amplitude should remain stable due to the presence of visual cues. This prediction is further motivated by evidence indicating reductions in auditory ERP amplitude in patients with noise induced hearing loss due to tinnitus (Attias et al., 1993) and in normal-hearing participants as noise thresholds change (Martin and Stapells, 2005; see also Woodward et al., 1990; Stevenson et al., 2012). Martin and Stapells (2005) observed that increased noise delivered via low pass filtering reduced auditory N1, N2, and P3 amplitudes, and also time-to-peak. Together, we predict that complementary cues provided by the visual signal in the AV condition should enhance recognition to a greater degree under lower S/N ratios.

Lip-movement typically precedes the auditory signal by tens of milliseconds in ecologically valid speech signals. Researchers have also argued that the leading visual speech cues provide predictive information that modulates early auditory encoding (e.g., van Wassenhove et al., 2005); effects of visual lead have been shown to facilitate auditory encoding, which is reflected in amplitude changes in the N1-P2 complex. We thus predicted that the N1 ERP amplitude associated with visual prediction would be greater for auditory-only stimuli vs. audiovisual stimuli in the high S/N ratio condition (e.g., Besle et al., 2004; van Wassenhove et al., 2005; Pilling, 2009). We also predicted that this difference between the audiovisual and auditory-only ERPs may be attenuated for lower auditory S/N ratios in which capacity increases.

### Capacity

The *null hypothesis* for capacity likewise predicts that integration will not change as a function of auditory S/N ratio within an individual listener. Incidentally, accuracy based models of integration often predict that each individual has a certain pre-established integration ability that does not change across listening conditions, contexts, or environments (Braida, 1991; Grant et al., 1998). To use one example, the Fuzzy Logical Model of Perception (FLMP; Massaro, 2004) predicts optimal integration of auditory and visual speech cues regardless of the perceived quality of the auditory and visual signals. This concept of optimality can perhaps best be translated in the capacity approach by assuming that optimal integration implies unlimited (or even super) capacity.

Our *alternative hypothesis* mirrors ERP hypotheses by predicting that capacity will be inefficient [*C*(*t*) <1] for high S/N ratios (clear signal), but become efficient [*C*(*t*) >1] for lower S/N ratios (−12 to −18 dB). Capacity should be limited in ideal listening environments since normal-hearing listeners do not normally utilize visual speech cues in such conditions. This is manifested in RTs by virtue of the fact that the AV distribution of RTs should not be much different than the auditory-only one (see Altieri and Townsend, 2011). Of course, as the auditory-only becomes slower in degraded conditions, the AV RT distribution becomes faster relative to the unisensory ones. The ERPs mirror capacity predictions because multisensory ERPs should fail to show evidence for visual gain (AV > A-only) in the clear listening condition. Hence, the EEG signal in the multisensory condition should not be sufficiently better than the one evoked by the auditory-only condition. These predictions are motivated by the law *of inverse effectiveness*, which stipulates that as auditory and visual-only recognition become less “effective,” AV integration improves relative to unisensory recognition speed/accuracy (e.g., Stein and Meredith, 1993; Stevenson et al., 2012). Likewise, cross-modal stochastic resonance (SR), similar to inverse effectiveness, predicts that the addition of noise to unisensory signals facilitates the detection of multisensory signals. However, SR differs from inverse effectiveness because it assumes that there is an optimal level of noise that can be added to a signal to achieve the maximum multisensory benefit (Ross et al., 2007; Liu et al., 2013).

## Methods

### Participants

Four young (age range of 20–28) right-handed native speakers of American English (1 female) were recruited from the student population at The University of Oklahoma. Participants reported normal or corrected to normal vision, and no participant reported having neurological or hearing problems. Participants were paid $8/h for their participation^1^. The Institutional Review Board at The University of Oklahoma approved this study.

This study obtained a sufficient number of data points to adequately estimate integrated hazard functions to compute robust capacity measures (Townsend and Nozawa, 1995), while also providing sufficient power to compare ERPs across conditions for each individual. Capacity and ERPs are time variable measures capable of showing differences in performance at different time points. Both capacity scores and analyses showing differences in ERPs will be displayed for each individual. Capacity also functions as a diagnostic tool for capturing information processing strategies at the level of the individual (e.g., Townsend and Nozawa, 1995; Townsend and Wenger, 2004; Townsend and Eidels, 2011; see Estes, 1956, for problems with averaging data). Our strategy should prove exceedingly useful for diagnosing audiovisual integration skills that can ostensibly vary as a function of auditory clarity, cognitive workload, or audiometric configuration, even within one individual (e.g., Altieri, 2010; Altieri and Townsend, 2011).

### Stimuli

The stimulus materials consisted of audiovisual full-face movie clips of two different female talkers. The stimuli were obtained from the Hoosier Multi-Talker Database (Sherffert et al., 1997). Two recordings of each of the following monosyllabic words were obtained from two female talkers: *Mouse, Job, Tile, Gain, Shop, Boat, Page*, and *Date*. These stimuli were drawn from a study carried out by Altieri (2010) and Altieri and Townsend (2011). The auditory, visual, and audiovisual movies were edited using Final Cut Pro HD 4.5. Each of the auditory files was normalized during the digitization process and sampled at a rate of 48 kHz (16 bits). Each movie was digitized and rendered into a 720 × 480 pixel clip at a rate of 30 frames per second. Video stimuli were played with a refresh rate of 60 Hz. The duration of the auditory, visual, and audiovisual files ranged from 800 to 1000 ms. White noise was mixed with each auditory file using Adobe Audition. This allowed for the creation of S/N ratios of −12 dB and −18 dB, in addition to a clear auditory S/N ratio in which noise was not mixed in with the stimuli.

The eight words in the stimulus set were presented in a total of seven blocks, including an AV-clear, AV−12, AV−18, A-clear, A−12, A−18, and V-only block. Each block consisted of 240 total trials, including 120 trials spoken by each talker. Each of the 8 words was presented a total of 30 times per block (15 spoken by each talker). In total, the experiment consisted of 1680 trials distributed over seven sessions within one 2-week period.

While the inclusion of a limited response set size was important for obtaining accurate RTs across a large number of trials and conditions, a potential disadvantage to this approach is that a closed stimulus set of 8 words lacks a degree of ecological validity. Listeners may process words differently compared to real world settings. For example, lip-reading accuracy scores will be higher for a set size of 8-monosyllabic words compared to a larger response set (Sumby and Pollack, 1954), or a sentence processing task (Altieri et al., 2011). One may object that the small set size encouraged listeners to recognize stimuli by relying on simple pattern recognition rather than word recognition. We remedied this by requiring participants to respond by pressing a button corresponding to the word they thought the talker said. The intent was to encourage listeners to engage in word recognition. This is in contrast to previous approaches which have required binary responses from participants to syllables (e.g., Massaro, 2004) or words (Winneke and Phillips, 2011). More importantly, if the words in our study were processed using pattern recognition based on simple auditory and visual features, it should be reflected in the capacity analysis. A preponderance of studies assessing the race model inequality using simple auditory or visual features, such as tones and dots (e.g., Miller, 1982; Berryhill et al., 2007) have consistently shown upper bound violations on independent race model predictions. When the upper bound on processing speed is violated, it indicates the presence of cross-modal dependencies and hence, a violation of independence. As discussed later, our pilot study using Gabor patches and auditory pure tones showed similar evidence for super capacity (as fast RTs) across each S/N ratio. This reflects a radically different profile from the capacity data in the word recognition experiment. Hence, the divergence in capacity results between simple auditory-visual detection and word recognition experiments indicates vastly different processing strategies—namely deeper processing for linguistic stimuli.

As a final caveat, noise was premixed with the stimuli prior to the experiment. Research indicates that participants may learn meaningless noise sounds over the course of many trials (e.g., Agus et al., 2010). However, our randomized block design, and the fact that each participant exhibited low accuracy scores in the low S/N ratio conditions (see Results), indicates that significant learning of noise patterns did not occur. Finally, while white noise may lack the properties of other masking strategies such as multi-talker babble that are most appropriate for sentence length materials (e.g., Bent et al., 2009), it still significantly reduces performance on vowel and consonant intelligibility (Erber, 2003).

### EEG recording

EEG recordings were made using EGI NetStation system with a 128-channel electrode net (Electro Geodesics International, Eugene, OR). Data were acquired continuously throughout the session and sampled at a rate of 1 kHz. The electrodes were referenced to the central (Cz) electrode. A significant advantage of using Cz as a reference electrode is that it is centrally located, and provides a reference that equal distances between electrodes on each hemispheres. The purpose was to obtain a central head location from which each frontal and parietal electrode could be referenced. Two electrodes, one located under each eye monitored eye movements, and a set of electrodes placed near the jaw were used for off-line artifact rejection. Channel impedances were maintained at 50 K Ohms or less for the entire testing session.

After down-sampling the data to 250 Hz, bad channels were identified and eliminated by visual inspection and ocular and other artifacts were removed automatically using EEGLAB V. 9 (http://sccn.ucsd.edu/eeglab/) with a statistical thresholding technique for detecting significant deviations. Baseline correction was carried out using an interval of 400 ms prior to the onset of the stimulus (i.e., word) in each condition (AV, A-only, and V-only). Data were organized into seven categories according to stimulus condition: AV (clear signal), AV (S/N ratio = −12 dB), Audiovisual (S/N ratio = −18 dB), A-only (clear signal), A-only (S/N ratio = −12 dB), A-only (S/N ratio = −18 dB), and V-only. The overall proportion of trials not rejected due to noise or artifacts per condition was over 0.90 for each condition [0.98 (AV clear), 0.94 (AV −12 dB), 93 (AV −18 dB), 0.98 (A clear), 0.93 (A −12 dB), 0.91 (A −18 dB), and 0.94 (V-only)]. Individual averages were computed at each time point for each electrode, with these averages computed for correct responses. All data were low-pass filtered at 55 Hz. A total of 36 electrodes (18 located on the frontal scalp region, and 10 located in the left parietal, and 8 in the left temporal scalp regions) were included in the data analysis. We selected a montage that included electrodes analyzed in previous studies, including left FC, C3, and CP (Pilling, 2009).

ERPs and times-to-peak-amplitudes for and participant were computed by obtaining the values of minima and maxima within specific time windows following stimulus onset. The primary peak ERP component of interest was the peak corresponding to phonological/language processing occurring roughly 400–700 ms post stimulus. Sometimes these peaks have been reported as being negative (depending on electrode positioning), although positive peaks connected to auditory language processing have been observed (e.g., Henkin et al., 2009; see Mehta et al., 2009, for discussion on the “P6” in word recognition). For the later ERP, we used the interval from 400 to 700 ms. We calculated positive peak amplitude values within this window that were significantly greater than 0, and the time to that peak using a maximum peak detection algorithm. For the N1 potential, we computed the minimum value in the trough (and time to negative peak) occurring between 70 and 120 ms post stimulus. The mean ERP value was calculated and submitted for analysis when the peak value for a given component differed significantly from 0 in an electrode.

### Procedure

Participants were seated at a fixed distance of 76 cm in front of a black and white CRT computer monitor with their chin placed on a chin rest. Experimental stimuli were presented using E-Prime version 2.0, and interfaced with NetStation software (EGI, Eugene OR) for the collection of continuous EEG recordings. Auditory stimuli were played via two speakers situated approximately 60 cm to the side.

Experimental trials began with a fixation cross (+) appearing in the center of the monitor followed after 200 ms by the stimulus. The stimuli were either auditory-only, visual-only or audiovisual trials, with each of these trials presented in separate blocks. Auditory stimuli were played at a comfortable listening volume (approximately 68 dB). Responses were collected via button press using the computer keyboard. Each of the buttons (1–8) was arranged linearly on the keyboard and was labeled with a word from the stimulus set. The labeling configuration was controlled across participants. Participants were instructed to press the button corresponding to the word that they judged the talker to have said, as quickly and accurately as possible. Responses were timed from the onset of the stimulus on each trial. Inter-trial intervals randomly varied on a uniform distribution between 750 and 1000 ms (from the time that the previous trial terminated once a response was detected). On auditory-only trials, participants were required to base their response solely on auditory information, and on visual-only trials participants were required to lip-read. Auditory-only trials were played with a blank computer screen. Likewise, visual-only trials were played without any sound coming from the speakers. The screen went blank once each trial containing a video was terminated. Each session consisted of one randomly ordered block per day and lasted approximately 45 min. To avoid order effects, the experimental blocks were randomized and presented in a unique order for each participant. Participants received 48 practice trials at the onset of each experimental block; data from these trials were not included in the subsequent analyses. Participants learned the keyboard response mappings during these practice trials such that head and eye movements were kept to a minimum.

## Results

### Behavioral analyses

The behavioral data were analyzed at two levels. First, mean accuracy and RT were examined across participants and auditory S/N ratios. This allowed for a coarse assessment of changes in integration efficiency as a function of S/N ratio. This method is less sensitive to fine gained temporal changes in efficiency relative to the analyses performed at the level of the distributions using the capacity coefficient (Townsend and Ashby, 1978, 1983; Wenger and Gibson, 2004).

Table 1 displays mean accuracy and RT results for each of the four participants, in addition to the mean and standard deviation (SD) across participants. The terms AV, A, and V denote the mean accuracy scores for the audiovisual, auditory, and visual conditions, respectively, while AV (RT), A (RT), and V (RT) denote the mean (SD) RTs in each of those conditions. Visual “Gain” (e.g., Sumby and Pollack, 1954; see also Grant, 2002; Bergeson and Pisoni, 2004) quantifies the relative benefit or the participants receives (in accuracy) by having the visual signal present in addition to the auditory signal. That is, what is the proportional gain in accuracy achieved by being able to see a talker's face? This is estimated as: *Gain* = [AV − A]/[1−A]; higher numbers indicate more efficient use of visual information, with 1 being the highest possible gain. In cases of extremely high unimodal accuracy, gain scores may become difficult to interpret. For example, Participant 4 showed a gain of −1.0, which results from a slightly lower AV relative to A-only score. However, both scores are effectively near ceiling, making the gain score of −1.00 meaningless in this case (in actuality, the data show an absence of gain). Visual gain in the temporal domain, labeled “Gain (RT),” signifies the overall benefit received in the RT domain from the presence of the visual signal. It is estimated as *Gain (RT)* = A(RT) − AV(RT). The proportion of auditory gain (gain afforded by the auditory speech signal over and above the visual) is also provided in the table *Gain_A* = [AV − V]/[1−V], as is the RT analogue *Gain_A(RT)* = V(RT) − AV(RT).

**Table 1:** **This table displays the mean accuracy scores for the audiovisual (AV), auditory-only (A), and visual conditions (V)**.

	**Sub. 1**	**Sub. 2**	**Sub. 3**	**Sub. 4**	**Mean**	**SD**
**(A) RESULTS FOR THE *CLEAR* AUDITORY SIGNAL CONDITION**						
AV	0.98	0.98	0.97	0.98	0.98	0.01
A-Only	0.98	0.98	0.97	0.99	0.98	0.01
V-Only	0.71	0.67	0.90	0.69	0.75	0.11
Gain	0.00	0.00	0.00	−1.00	−0.25	0.50
Gain_A	0.93	0.94	0.67	0.94	0.87	0.13
AV (RT)	1455 (310)	1586 (286)	1273 (324)	1272 (413)	1397	153
A (RT)	1466 (585)	1583 (456)	1253 (267)	1280 (255)	1396	157
V (RT)	1705 (464)	1946 (458)	1405 (291)	1771 (472)	1707	225
Gain (RT)	11	−3	−20	8	−1	12
Gain_A (RT)	250	360	132	499	310	157
**(B) RESULTS FOR THE *−12 dB* AUDITORY CONDITION**						
AV	0.93	0.93	0.90	0.95	0.93	0.02
A-Only	0.72	0.73	0.69	0.69	0.71	0.02
V-Only	0.71	0.67	0.90	0.69	0.75	0.11
Gain	0.75	0.74	0.68	0.84	0.75	0.07
Gain_A	0.76	0.79	0	0.84	0.60	0.40
AV (RT)	1263 (344)	1174 (521)	1361 (341)	1296 (244)	1274	78
A (RT)	1966 (778)	2271 (572)	1604 (464)	1958 (625)	1950	273
V (RT)	1705 (464)	1946 (458)	1405 (291)	1771 (472)	1707	225
Gain (RT)	703	1097	243	662	676	349
Gain_A (RT)	442	772	44	475	433	299
**(C) RESULTS FOR THE *−18 dB* AUDITORY CONDITION**						
AV	0.94	0.90	0.95	0.89	0.92	0.03
A-Only	0.33	0.33	0.30	0.36	0.33	0.02
V-Only	0.71	0.67	0.90	0.69	0.75	0.11
Gain	0.91	0.85	0.93	0.83	0.88	0.05
Gain_A	0.79	0.70	0.50	0.65	0.66	0.12
AV (RT)	1365 (253)	1708 (199)	1331 (269)	1384 (345)	1447	175
A (RT)	2223 (786)	2748 (795)	1938 (605)	2076 (577)	2246	354
V (RT)	1705 (464)	1946 (458)	1405 (291)	1771 (472)	1707	225
Gain (RT)	859	1040	607	692	800	191
Gain_A (RT)	340	238	74	387	260	139

The Gain scores {[AV − A]/[1 − A] & [AV − V]/[1 − V]} and RT Gain scores {(ART − AVRT) & (VRT − AVRT)} are shown as well.

Results from the clear auditory condition are shown in Table 1A, the −12 dB condition in Table 1B, and the −18 dB in 1C. On average, identification accuracy in the visual-only condition was 75% with the three out of four participants scoring ~70% and one scoring 90%. This observation was consistent with previous findings in an 8-alternative forced-choice task (Sumby and Pollack, 1954; Altieri and Townsend, 2011).

The results in Table 1A reveal virtually no difference in accuracy between the AV and A condition across subjects. Not surprisingly, Gain scores were close to 0 for each participant in the clear condition. The RT analyses revealed little difference between audiovisual and auditory trials; RT Gain scores were near zero, revealing that the visual signal failed to facilitate processing in the temporal domain. Overall, the RT results suggest that audiovisual integration either did not occur in this condition, or possibly that it either did not provide any benefit or extract any cost.

In the −12 dB S/N condition (Table 1B), recognition accuracy in the audiovisual condition was higher than in the auditory-only condition. Gain scores for each participant were approximately 70% or greater, with an overall mean of 75%. Similarly, a noticeable visual gain was observed in the RT data, with AV RTs being nearly 700 ms faster on average compared to auditory-only RTs. This level of gain was statistically greater than that observed in the clear condition [*t*_(3)_ = 3.9, *p* < 0.05].

The −18 dB S/N ratio condition (1C) revealed a pattern of results similar to the −12 dB S/N ratio. Auditory-only recognition accuracy was considerably above chance for each participant although performance in all of the conditions was extremely degraded. Nonetheless, audiovisual recognition accuracy (mean 92%) was markedly higher compared to auditory-only accuracy (mean 33%). Consequently, proportional Gain scores were significantly higher in the −18 compared to the −12 dB condition [*t*_(3)_ = 4.3, *p* < 0.05]. Interestingly, accuracy scores in the audiovisual, auditory-only, and visual-only conditions were consistent with those observed in previous word identification studies using 8-alternative forced-choice tasks and identical S/N ratios (e.g., Sumby and Pollack, 1954). Visual gain in the RT data, under the most degraded listening conditions, was also significantly greater compared to the gain scores in clear listening condition [*t*_(3)_ = 28, *p* < 0.001], but not compared to the −12 dB condition. Taken together, mean accuracy and RT results indicate that the most efficient integration occurs between −12 and −18 dB, and that integration may not need to occur when listening conditions become less degraded due to ceiling effects in the auditory modality (see Ross et al., 2007).

#### Capacity and integration efficiency

**Figure 2:**
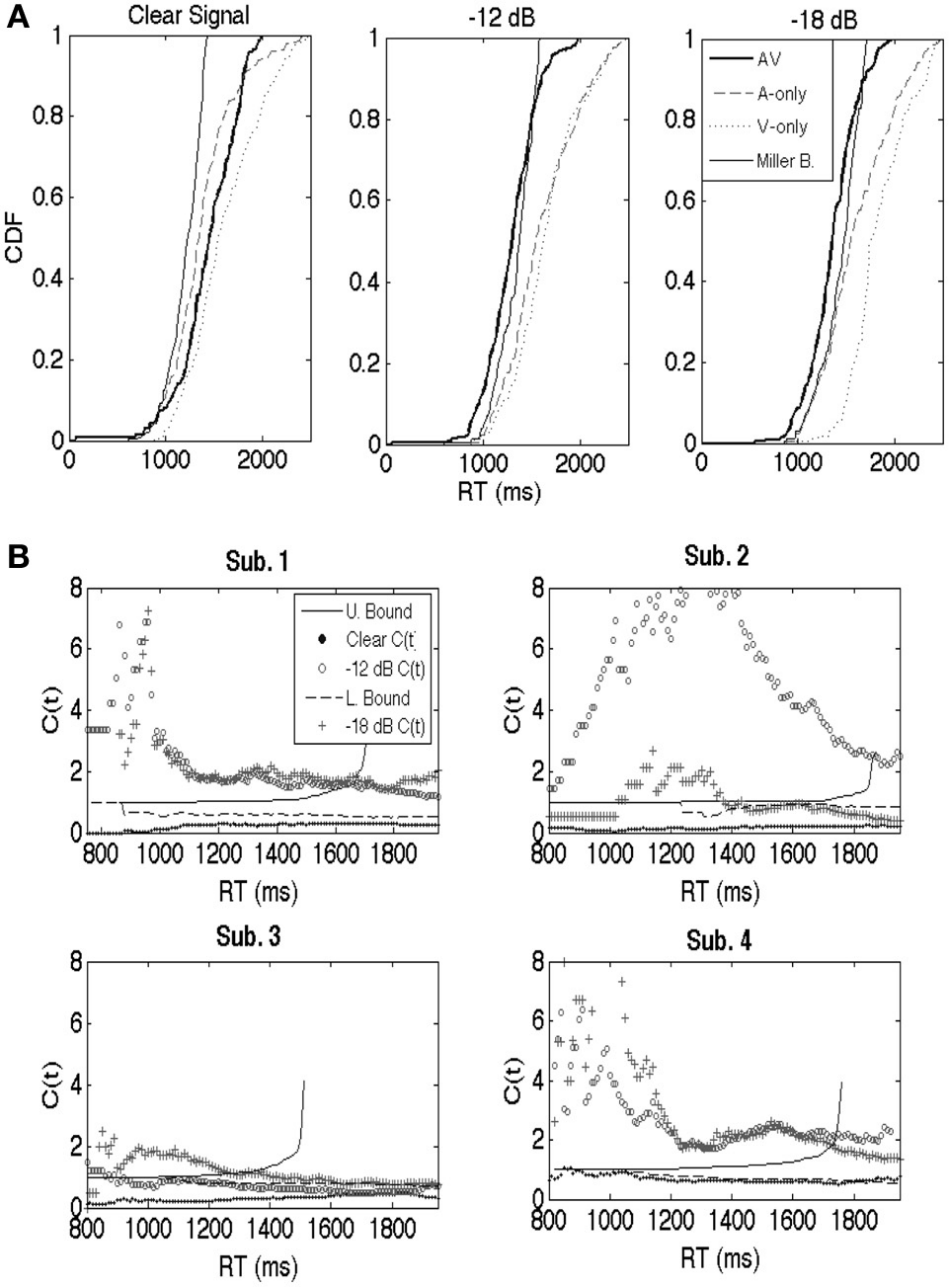
**(A)** For purposes of illustration, cumulative distribution functions (CDFs; participant 4) are shown separately for the auditory-only, visual-only, and audiovisual trials for the clear, −12 dB, and −18 dB listening conditions. Miller and Grice bounds are plotted as well. **(B)** Similar to the CDFs, capacity is a continuous function. Results are shown separately for participants 1 through 4. Participant 1's results are shown in the upper left, 2's in the upper right, 3's in the lower left, and 4's in the bottom right. Each panel displays *C(t)* across each listening condition, as well as the upper (Miller) and lower (Grice) bounds translated into capacity space (cf. Townsend and Eidels, 2011 for equations). The symbols (clear, −12 dB, and −18 dB) denote the values for the capacity function. Note that there should be separate upper and lower for each auditory S/N ratio. Our strategy was to plot the averaged bounds to avoid clutter, although interpretations of the data would not have changed regardless of the bounds used.

Figure 2A shows example cumulative distribution functions (Participant 4) obtained from the audiovisual, auditory, and visual-only conditions. Results are shown across each S/N ratio. The Miller bound [F_A_(t) + F_V_(t)] was violated across several time points in the lower S/N ratio conditions. Interestingly, F_AV_(t) was less than F_A_(t) (the fastest unimodal condition) across most time points, indicating lower Grice bound (Grice et al., 1984) violations (Grice bound = max{F_A_(t), F_V_(t)}). The Grice bound sets a lower limit on independent processing, where violations suggest negative inter-modal dependencies, and inefficient integration.

The capacity coefficient, *C(t)*, was calculated for each participant across the three listening conditions (clear, S/N = −12 dB, S/N = −18 dB). Capacity function values are plotted as symbols in Figure 2B (correct responses were used in the capacity calculations). Capacity analyses were computed by pooling RT data across the 8 words in the computation of the cumulative hazard functions shown in Equation 1. While pooling data across stimuli with different onset consonants (e.g., “b” vs. “sh”) may obscure effects for individual words, the same overall trend was observed across each stimulus (see **Appendix**). A greater audiovisual benefit, in terms of both mean RT and accuracy, was observed for each stimulus in the −12 and −18 dB conditions. Hence, mean RTs were considerably faster, and mean accuracy was also greater in the audiovisual condition compared to either the auditory or visual-only conditions. Conversely, none of the stimuli showed evidence consistent with an audiovisual benefit in the “clear” condition, just as expected.

The capacity results in Figure 2B followed a similar pattern across participants: limited capacity in the “clear” condition, and efficient integration marked by violations of the Miller bound, at least at some time points, in more difficult listening conditions. The upper or Miller Bound is depicted by the solid line and represents an upper limit on independent race model predictions. Violations of these bounds in the −12 and −18 dB conditions strongly suggests violations of independence, and hence, facilitatory cross-modal dependencies. The *C(t)* results for the clear listening condition hover well below 1 and near 1/2 across nearly all time points and participants. Consequently, *C(t)* violated the lower bound in every participant for at least a few time points. The All of this serves to clarify the ambiguity with respect to integration obtained from the mean data. Recall that those data suggested either inefficient on non-existent integration. The capacity coefficients clearly show that the integration was in fact extremely inefficient. The lower bound represents a lower limit on independent race model predictions and is represented by the dashed line in each panel^2^. The *C(t)* data for each participant in the −12 dB and −18 dB S/N ratios showed consistent violations of the upper bound, particularly for early response times. Although the results revealed individual differences (i.e., lower efficiency for Participant 3, and higher capacity in the −12 dB than the −18 dB condition for Participant 2), the qualitative pattern of results held across participants.

Thus, the results show rather strong evidence in favor of the predicted pattern: inefficient audiovisual integration under optimal listening conditions but highly efficient integration under degraded listening conditions. The ubiquitous violations of the upper and lower bound strongly suggests facilitatory interactions in the case of upper bound violations, and inhibitory interactions in the case of lower bound violations (e.g., Eidels et al., 2011). As shown in Figure 1, interactive models with separate decisions on the auditory and visual modalities can account for such violations via interactive mechanisms across channels that change from inhibitory to excitatory as a function of the clarity of the auditory signal. Such an account is consistent with the idea that extensive uni-sensory processing takes place in auditory and visual pathways, and that interactions occur even in the later stages of recognition (Bernstein et al., 2004; Ponton et al., 2009; Naue et al., 2011).

### ERP analysis

#### Late peak

**Figure 3:**
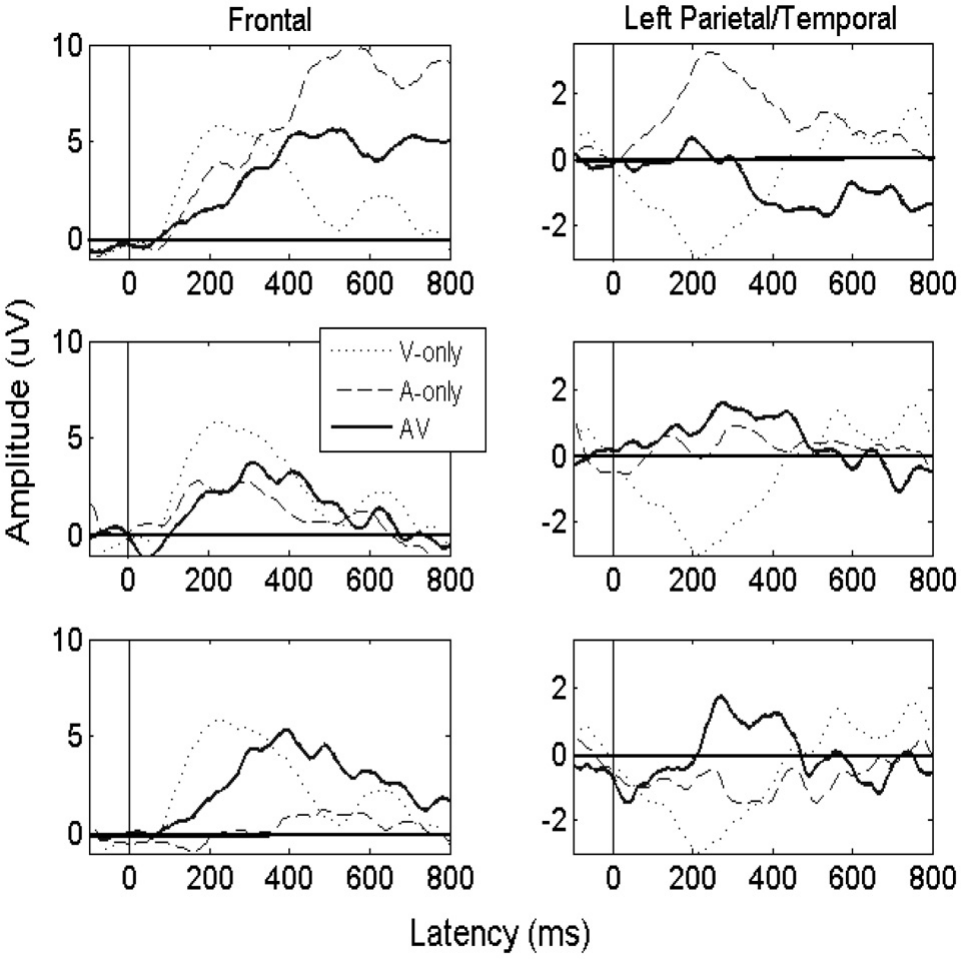
**Figure displaying averaged ERPs obtained from the “clear” auditory condition (top), the −12 dB S/N ratio (middle), and the −18 dB S/N ratio (bottom).** The solid line shows the averaged ERPs for the audiovisual (AV) condition, and the dashed line represents the auditory-only (A) condition. The dotted line represents the visual-only (V) condition, which is strongly expressed in frontal regions due to feed-forward connections originating from occipital brain regions. Results are shown using sample electrodes from the frontal scalp region (e.g., F3, F7, and Fz), and Parietal/Temporal Region (e.g., C3, P3, and T5).

Figure 3 displays averaged ERPs calculated across electrodes from the frontal and parietal/temporal scalp regions for purposes of illustration. Results are shown from audiovisual (AV) auditory-only (A) and visual-only (V) ERPs across three S/N ratios. ERPs were smoothed using a moving window approximation. ERP amplitudes were determined within an electrode by utilizing a function that computed the maximum peak value within the time window. First, we utilized a time window ranging from 400 to 700 ms when determining the peak value, and also the latency at which it occurred. We can observe that peaks emerged in the audiovisual condition, on average, in the 400-500 ms time window. While the late peak was generally reduced in the low S/N auditory-only conditions, significant potentials did emerge post 400 ms, highlighting the importance of analyzing later potentials in language perception studies. A positive visual evoked potential (~200 ms) was also observable across frontal electrodes, and a negative potential due to a polarity reversal was observed in temporal/parietal electrodes. The auditory and audiovisual amplitudes were generally positive across anterior and posterior scalp regions, although one may observe that the auditory potentials were considerably attenuated, even to the point of becoming slightly negative for later times, as noise increased. The quantification procedure of using positive peak was consistent for both frontal and temporal/parietal sites.

**Figure 4:**
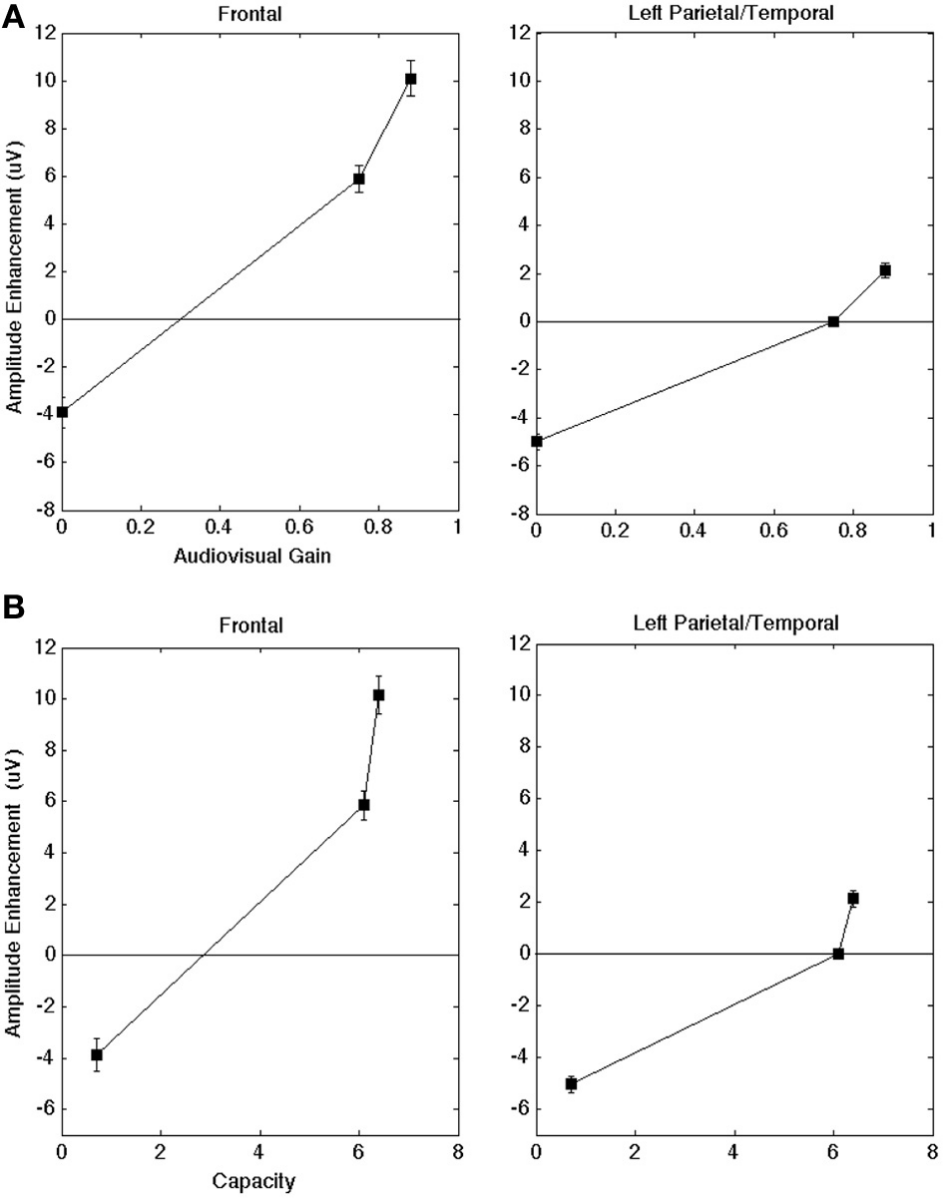
**(A)** AV gain in amplitude for the peak ERP (AV − A) as a function of audiovisual gain across brain regions of interest. A positive value means that the average AV amplitude was larger than the A. The scores are collapsed across each of the four participants. **(B)** Audiovisual gain in amplitude as a function of capacity scores (in the clear, −12 dB, and −18 dB S/N ratio conditions, respectively) across brain regions of interest. The scores are collapsed across each of the four participants. Error bars denote one standard error of the mean computed across individual electrodes (across subjects) within a given region.

For the initial statistical analysis on the late amplitude, we carried out a One-Way ANOVA on individual electrodes across all participants to determine whether there was a main effect for modality (α levels were 0.05 unless otherwise indicated). The ANOVA on the aggregate data indicated a significant effect for modality. Figure 4A plots results across the frontal and left parietal/temporal regions (AV − A) for the ERP as a function of audiovisual gain. All error bars represent one standard error of the mean peak amplitude calculated across individual electrodes. In order to illustrate the quantitative relationship between capacity values and ERPs, Figure 4B displays the ERP enhancement scores as a function of capacity obtained in the clear, −12 dB, and −18 dB listening conditions, respectively. Maximum capacity values were obtained for each auditory S/N ratio across the intervals displayed in Figure 2 and averaged across individual observers (e.g., Altieri and Townsend, 2011). Figure 4 shows that as audiovisual gain and capacity increased the difference between the audiovisual and the unisensory signals also increased.

In both frontal and left regions, visual speech significantly enhanced the late ERP amplitude [*F*_(2, 629)_ = 3.3, *p* < 0.05] compared to the auditory and visual-only ERPs. This significant differences in multisensory vs. unisensory ERPs suggests that changes in amplitude were not merely the superposition of component effects (in which the AV peak amplitude would equal the sum of the A and V-only peaks, AV = A + V). The ANOVA testing the interaction between region and modality was significant, indicating that the strongest effects occurred in frontal regions compared to left parietal/temporal regions [*F*_(4, 629)_ = 2.8, *p* < 0.05]. The interaction can be observed in Figures 4A,B, which shows greater ERP amplitude increase the frontal region compared to the left regions. The observed interaction, and the fact that the changes in amplitude were not merely the superposition of component effects (where the AV peak amplitudes simply reflect the sum of the auditory-only and visual-only peak amplitudes, AV = A + V). The ERP analysis also evidenced significant enhancement compared to the auditory-only (AV vs. A) ERP peak [*t*_(629)_ = 2.2, *p* < 0.05]. These findings appear contrary to previous literature indicating that the presence of visual speech in the AV condition should yield a reduction rather than enhancement in peak amplitude (e.g., van Wassenhove et al., 2005; Pilling, 2009; Winneke and Phillips, 2011).

The reason for the observed discrepancies likely lies in the fact that audiovisual integration mechanisms operate differently across listening conditions. Previous studies [e.g., van Wassenhove et al. (2005) and Pilling (2009)] analyzed the N1/P2 ERP components under clear auditory listening conditions. Interestingly, the contrasts for the different S/N ratio conditions support this hypothesis. The AV_High_ vs. A_High_ contrast in the mean ERP (clear condition) yielded the predicted reduction in the audiovisual peak amplitude [*t*_(631)_ = −3.2, *p* = 0.001]. Next, the contrast for the AV_Low_ vs. A_Low_ showed strong evidence for AV enhancement (i.e., AV > A) [*t*_(629)_ = 5.1, *p* < 0.001], although the AV_Med_ vs. A_Med_ only showed evidence for a non-significant trend (*p* = 0.11) toward AV enhancement.

The results for the *t*-test for each of the four individual participants are shown in Table 2,^3^. The key analyses for each participant included *t*-tests assessing the overall AV − A contrast on peak amplitude (across all frontal and parietal/temporal electrodes for the observer), and the contrasts for the high, A_High_V − A_High_, medium A_Med_V − A_Med_, and low A_Low_V − A_Low_ S/N ratio experimental conditions. Participants 2, 3, and 4 showed evidence for audiovisual enhancement (AV − A >0). Participant 1's results diverged from the other 3 participants in that an overall audiovisual reduction rather than enhancement was observed in the lowest S/N ratio listening condition. Frontal regions showed a significant reduction in the −18 dB condition. However, in the left parietal/temporal scalp regions, reduction was observed in the high S/N ratio while enhancement was observed in the −18 dB condition {the interaction between region and condition was significant [*F*_(7, 144)_ = 14.1, *p* < 0.001]}.

**Table 2:** **This table displays contrast results for the ERP peak amplitude for Participants 1 through 4**.

**Contrast**	**N1**	**Late ERP**
**SUB. 1**		
AV − A	2.30^*^	−1.03
High	1.10	1.17
Medium	2.70^**^	−0.21
Low	0.88	−4.10^***^
**SUB. 2**		
AV − A	2.40^*^	0.49
High	0.41	−4.20^**^
Medium	3.20^**^	0.70
Low	0.80	3.60^***^
**SUB. 3**		
AV − A	1.90	4.40^***^
High	0.89	2.70^**^
Medium	3.60^***^	0.31
Low	1.50	3.60^***^
**SUB. 4**		
AV − A	2.45^**^	0.50
High	0.90	−4.20^***^
Medium	3.90^***^	0.70
Low	1.10	3.60^***^

Signed numerical t-values and significance (

*** p < 0.001;

** p < 0.01; and

* p < 0.05) are shown in each cell corresponding to the high, medium, and low listening condition.

#### N1 component

We now briefly summarize data from the N1 component to bolster claims showing evidence for early audiovisual interactions during encoding (e.g., Besle et al., 2004; van Wassenhove et al., 2005; Pilling, 2009; Stevenson et al., 2012). A small negative amplitude (N1 ~70–120 ms) was observed in the audiovisual conditions, and sometimes in the auditory-only. One reason why the early AV amplitude may have been similar to the A-only is that the visual signal essentially failed to provide useful bottom-up sensory information (although cf. van Wassenhove et al., 2005). However, under the medium (−12 dB) listening conditions, the visual signal likely provided early bottom-up sensory input that could eventually be combined with the degraded auditory signal. Interestingly, the results suggest that the N1 amplitude of the AV signal was once again reduced relative to the A-only in the −18 dB condition. Our preliminary explanation is that when the auditory signal became sufficiently degraded, the visual signal once again failed to provide sufficiently bottom-up sensory support. Nonetheless, as processing progressed, auditory phonemic information could be effectively extracted and integrated with visual cues (as observed by increased capacity and enhancement of the later ERPs). Of course, there exist S/N ratios in which the auditory signal becomes so degraded that the visual signal fails to be of any benefit (see Sumby and Pollack, 1954; Ross et al., 2007).

For the statistical analyses, the ANOVA testing the interaction between region and modality was significant [*F*_(4, 508)_ = 14.1, *p* < 0.001]. This indicates that a greater negative peak amplitude (in the AV vs. A-only) occurred in the frontal compared to the left regions, mainly in the −12 dB condition. Individual contrasts for the N1 are also shown in Table 2. These data point to multisensory enhancement for Participants 1 through 4 in the −12 dB S/N (and an overall enhancement in Participants 1, 2, and 4 driven by the −12 dB condition). Although results diverged from previous findings showing AV suppression, our ERP results are in agreement with previous literature showing that the visual signal interacts with the auditory neural processing during early attentional and encoding stages. The difference in our task and previous studies employing discrimination with short matched/mismatched consonants (e.g., van Wassenhove et al., 2005) may help account for observed differences in early components.

#### Time-to-peak analysis

**Figure 5:**
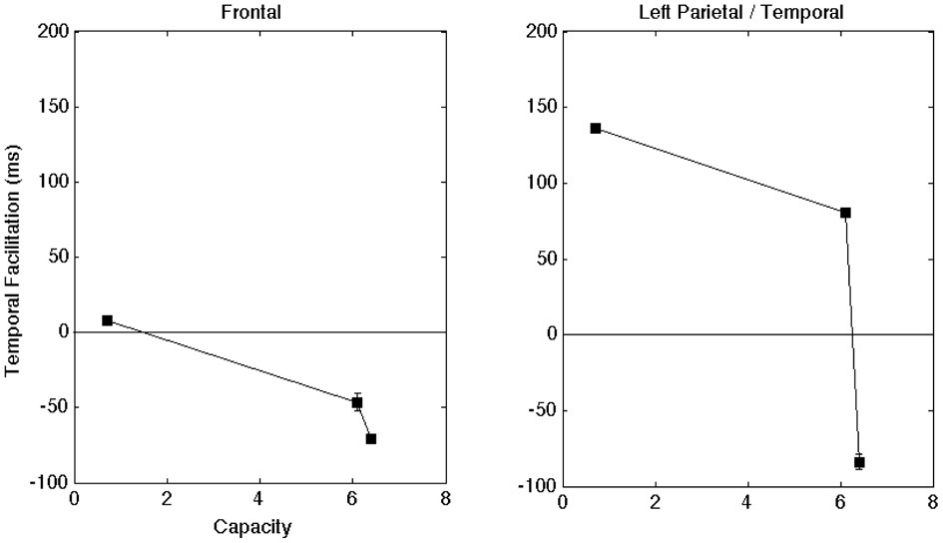
**Shows the latency differences in the ERP peak component plotted as a function of capacity for the frontal and left regions.** A positive value means that the time-to-peak was faster in the AV compared to the A-only condition. Once again, error bars denote one standard error of the mean computed across individual electrodes (across subjects) in a given region.

The time-to-peak analyses were less consistent across participants, but they still provided intriguing insights. Once again, we carried out one-way ANOVAs (α = 0.05) using data obtained from individual electrodes across participants. The results from the combined data analysis on the late ERP for time-to-peak-amplitude demonstrated significant effects for modality. First, the presence of visual speech contributed to an overall slowdown in the time-to-peak for the late ERP [*F*_(2, 539)_ = 4.9, *p* < 0.01]. The *t*-tests for difference of means from the AV − A contrasts (α = 0.01) showed evidence for slower audiovisual processing when the auditory S/N ratio was clear/high [*t*_(558)_ = 5.8, *p* < 0.001], but facilitation when the auditory S/N ratio was −18 dB [*t*_(558)_ = −4.3, *p* < 0.001]. Interestingly, an examination of the AV_Time_ − A_Time_ contrasts across listening conditions showed evidence for a slowdown in AV time-to-peak in the clear listening condition [*t*_(339)_ = 2.6, *p* = 0.01], and a trend in the same direction for the −12 dB S/N ratio [*t*_(339)_ = 2.3, *p* = 0.02]. Figure 5 shows the temporal facilitation effects for the frontal and left parietal/temporal regions (AV_Time_ − A_Time_) as a function of reaction time (RT) gain.

The time-to-peak contrasts (AV_Time_ − A_Time_) for each participant are shown in Table 3. First, Participant 1 showed evidence for audiovisual temporal slow-down in the time-to-peak measurement in the clear listening condition, although the data for Participants 2 and 4 showed evidence for facilitation. Conversely, Participants 2, 3, and 4 showed evidence for temporal slow-down in either the −12 or −18 dB conditions. This analysis broken down by individual subjects data supports to the hypothesis that cross-modal interactions occur in the later stages of integration as phonetic and word recognition unfold (e.g., van Wassenhove et al., 2005; Ponton et al., 2009).

**Table 3:** **Table displaying contrast results for the time to peak for each participant**.

**Contrast**	**Late ERP**
**SUB. 1**	
AV − A	−0.42
High	2.90^**^
Medium	−1.30
Low	−1.16
**SUB. 2**	
AV − A	−1.85
High	−2.90^***^
Medium	0.77
Low	3.90^***^
**SUB. 3**	
AV − A	0.60
High	0.55
Medium	2.50^*^
Low	−0.66
**SUB. 4**	
AV − A	−1.85
High	−5.90^***^
Medium	−0.77
Low	3.90^***^

Negative signs indicate a faster AV relative to A-only time to peak (AV − A), whereas a positive number indicates faster A compared to AV time to peak. Significance (again,

*** p < 0.001;

** p < 0.01; and

* p < 0.05) is shown in each cell corresponding to the high, medium, and low condition.

In summary, the accuracy, capacity, and ERP results provide converging evidence that poorer listening conditions afford the greatest efficiency in audiovisual integration. These results suggest that visual information influenced neural integration processes and were responsible for the observed effects on ERP peak amplitudes.

## General discussion

The purpose of this study was to assess integration efficiency under different listening conditions while investigating how efficiency relates to brain activity. We proposed that capacity represents a continuous measure of efficiency (e.g., Townsend and Nozawa, 1995; Townsend and Wenger, 2004). This approach assumes that word recognition occurs as soon as either auditory or visual information (corresponding to a specific word/category) reaches a threshold (see Altieri and Townsend, 2011). This study represents an approach that associated ERPs with a framework that makes testable and statistically motivated predictions. As a corollary, this framework provides a mechanism to account for capacity changes and co-varying changes in ERPs across listening environments (i.e., facilitatory/inhibitory cross-modal connections during language perception; Altieri and Townsend, 2011; Eidels et al., 2011).

To review, independent models assume that auditory and visual information does not interact as recognition unfolds. Independence predicts that processing capacity/integration efficiency should be approximately equal to 1 across S/N ratios. Violations of independence produced by facilitatory cross-modal interactions elicit a level of efficiency that is greater than 1 (violating the upper bound), while inhibitory interactions yield levels markedly less than 1, and can even approximate fixed capacity [i.e., *C(t)* = 1/2] (Townsend and Wenger, 2004; Eidels et al., 2011; see also Townsend and Nozawa, 1995). A unique feature of capacity is that one can show evidence for different levels of work completed and therefore differences in energy expenditure across time and listening conditions. This differs from other frameworks which conceptualize integration efficiency as an invariant construct unique to a given individual (e.g., Grant et al., 1998; Massaro, 2004).

The prediction for the late ERP peak component was hypothesized to follow the same qualitative pattern as capacity in the behavioral/RT domain. This should occur due to the low availability of auditory information under degraded listening conditions, which allows complementary visual information to increasingly assist auditory recognition (e.g., Grant et al., 1998; Erber, 2003). Thus, as integration efficiency increases under degraded conditions, the peak amplitude should increase in the audiovisual condition relative to the auditory-only condition (AV > A). We predicted that the neural predictions would covary with the capacity predictions due to the fact that ERP activity may be associated with synchronous neural firing patterns. This is especially true as the A-only amplitude decreases when less phonemic information about manner of articulation is available in the auditory signal. ERP hypotheses were also motivated by findings showing that visual information influences early auditory encoding (e.g., van Wassenhove et al., 2005) and more significantly, the stages of language processing (e.g., Arnal et al., 2011). In a study using magnetoencephalography (MEG), Arnal et al. (2011) (see also Arnal et al., 2009) showed that valid or otherwise congruent audiovisual speech signals were associated with a correlation between a late ERF and an increase in delta frequencies (3–4 Hz). The time-course and MEG scalp topographies indicated that these effects occurred in regions associated with higher language processing. Increasingly useful visual information in lower S/N ratios should lead to more efficient use of visual information in terms of capacity, which ought to be associated with a corresponding increase in a neural index of integration.

Also recall that SR is similar to inverse effectiveness, but differs inasmuch as it assumes that there is an optimal level of noise for achieving maximum multisensory gain. This makes sense in the context of speech perception; if the auditory signal becomes too degraded as discussed previously, then multisensory perception will begin to approximate visual-only performance which is often quite poor (because the auditory signal fails to contain any useful information; Ross et al., 2007; Liu et al., 2013). In a multisensory word recognition task, Liu and colleagues found that the optimal level of AV gain [AV − A]/[1−A] occurred at −12 dB rather than lower S/N ratios. The AV peak amplitude for the time range of 130–200 ms also showed the highest degree of multisensory benefit in the −12 dB condition. One reason we may have observed the highest level of audiovisual gain under the −18 rather than −12 dB condition is that we used a smaller set size of 8 words, which constrained task difficulty.

### Summary of findings

Integration efficiency was universally inefficient for the high S/N ratio [*C(t)* <1] but efficient across lower S/N ratios [*C(t)* > 1] (−12 to −18 dB) as predicted.^4^ Contrary to intuition, this suggests that multisensory integration may not always be beneficial, particularly for normal-hearing listeners in quiet listening environments. Violations of independent predictions may be observed in the violation of the lower and upper bounds, respectively, in Figure 2B. This relation held for each of the four participants. The corresponding audiovisual gain scores [AV−A]/[1−A] and RT gain scores (A_RT_ − AV_RT_) also demonstrated a consistent pattern across each participant, increasing as auditory S/N ratio decreased. These results corroborated recent findings demonstrating the utility and methodological advantages of utilizing capacity as a fine-grained, model-theoretic measure of integration efficiency in speech perception (Altieri, 2010; Altieri and Townsend, 2011). The violations of independence between audiovisual processing as evidenced by *C(t)* should be somewhat unsurprising given the preponderance of evidence for audiovisual interactions in accuracy (e.g., Massaro, 1987, 2004) and mean ERPs (e.g., Besle et al., 2004; van Wassenhove et al., 2005, 2007; Ponton et al., 2009; Winneke and Phillips, 2011).

Results further demonstrated that as visual information became more useful in lower auditory S/N ratios, capacity values co-varied with significant audiovisual enhancement (AV > A) in the late ERP. These data are consistent with the prediction that visual signals enhance auditory processing in both behavior and in neural processing. An alternative interpretation of this finding is that as auditory noise increases, neural processing as reflected by the EEG increases due to processing difficulty. We argue for the former position because the behavioral and neural data are consistent with the predictions of stochastic resonance (SR). In the clear condition, the average A-only ERP peak was robust, but becomes increasingly attenuated as the auditory signal is degraded by noise. This finding is consistent with ERP research showing evidence for decreased auditory amplitude as noise and processing difficulty increases (Attias et al., 1993; Stevenson et al., 2012). However, when visual information complements the auditory signal, the AV ERP shows a gain relative to the low S/N ratio A-only ERP, thereby reflecting auditory processing in the clear condition.

Finally, the time-to-peak analysis (i.e., AV_Time_ − A_Time_ >0) provided additional support for the hypothesis that visual information interacts with the processing of auditory speech, although the results were somewhat less consistent. The presence of visual speech information is known to speed up auditory processing under different listening conditions (see van Wassenhove et al., 2005). Interestingly, the time-to-peak analysis even showed evidence for inhibition for some participants (i.e., AV_Time_ > A_Time_) in the ERPs when the auditory S/N ratio was lowest (−18 dB). The observed audiovisual slow-down in the time-to-peak analysis presents an intriguing finding. On one hand, degraded listening conditions yield better integration, both in terms of accuracy and capacity, and converging neural evidence was also observed in the ERP analysis. On the other hand, degraded listening conditions led to an audiovisual slow-down in terms of the ERP time-to-peak in three participants. The reason for this dissociation is currently unclear. This slowing down of processing in the neural domain may be accounted for by the lack of auditory evidence and the reliance on visually based internal predictions (see van Wassenhove et al., 2005). Since the increase in capacity at lower S/N ratios appears to result from the recruitment of additional resources, it is plausible that the increase in time-to-peak could be due to the cost associated with obtaining extra resources. Crucially, the results from the ERP peak and time-to-peak analyses show evidence for a combined increase in AV amplitude for later processing times in the higher capacity condition relative to the lower capacity conditions. A calculation of the ratio of AV peak amplitude to time-to-peak further indicates that there is more amplitude per unit of time in the highest capacity condition (mean = 15.8) relative to the low (mean = 33.1) [*t*_(142)_ = 5.9, *p* < 0.00001]. These effects were marginally significant for the lowest capacity condition (i.e., Clear) vs. the −12 dB condition (mean = 18.7) [*t*_(142)_ = 1.65, *p* = 0.10].

Our findings constitute a significant development by revealing a close correspondence between integration efficiency, as measured by behavior on one hand, and brain signals on the other. Hence, we now have converging evidence for interactive processing in audiovisual speech perception. In this framework (Figure 1), auditory and visual information undergo unisensory processing in primary sensory cortices, although linguistic recognition can be enhanced or inhibited via cross-modal connections. Specifically, our combined capacity and neural analysis indicate that these crucial cross-modal interactions occur in later stages during conscious language perception.

### Conflict of interest statement

The authors declare that the research was conducted in the absence of any commercial or financial relationships that could be construed as a potential conflict of interest.

## Footnotes

^1^These same subjects participated in a non-speech pilot study for three blocks of 800 trials over a period of 3 days. The study involved presenting visual stimuli (Gabor patches), auditory pure tones presented at three auditory S/N ratios: clear, −12 dB, and −18 dB), and simultaneously presented (AV) Gabor patches and auditory pure tones, in addition to catch trials consisting of white noise and a blank screen. Participants were required to make a “yes” response by pressing the right button on the mouse if a Gabor patch appeared on the screen, they heard an auditory tone, or saw both a Gabor patch and auditory tone. They were required to respond “no” by pressing the left mouse button on blank catch trials. As in the primary experiment, processing capacity was computed for each auditory S/N ratio, and EEG recordings were obtained via a dense electrode 128-channel net.

^2^Townsend and Eidels (2011) translated the upper Miller bound, and lower Grice bound into a unified capacity space. Figure 2B depicts these bounds in each of the four panels.

^3^The α level was set to the more conservative level of 0.01 to adjust for multiple comparisons.

^4^Limited capacity could also result from another situation where the auditory signal-to-noise ratio is low enough that auditory-only recognition accuracy approximates floor performance. Of course, the mechanisms contributing to capacity limitations would be different from cases where auditory recognition is near ceiling. We would probably not implicate cross-channel inhibition as a causal mechanism underlying capacity limitations in such cases. Under this scenario, recognition would be functionally visual-only, where the audiovisual speed and accuracy would probably not differ significantly from the visual-only condition. Some studies of audiovisual gain t have found that efficiency reaches a peak around −12 to −18 dB before tapering off at lower auditory S/N ratios (e.g., Ross et al., 2007).

## Appendix

**Table:**

	**Boat**	**Date**	**Gain**	**Job**	**Mouse**	**Page**	**Shop**	**Tile**
**MEAN RTs**								
AV_High	1241	1256	1254	1236	1367	1335	1274	1209
AV_Med	1232	1255	1214	1203	1180	1241	1234	1260
AV_Low	1396	1420	1453	1539	1491	1476	1454	1431
A_High	1280	1286	1186	1295	1275	1262	1212	1229
A_Med	1647	1636	1756	2010	1600	2006	1825	1725
A_Low	1853	1888	1953	2070	1893	1988	1948	1993
V-only	1566	1584	1676	1694	1749	1689	1790	1644
**ACCURACY**								
AV_Med	1.0	0.97	0.97	0.73	0.97	1.0	0.97	0.93
AV_Low	0.97	0.97	0.97	0.64	0.97	0.97	0.97	0.97
A_Med	0.63	0.57	0.78	0.57	0.87	0.82	0.83	0.65
A_Low	0.32	0.25	0.43	0.25	0.23	0.55	0.33	0.29
V_Only	0.93	0.76	0.91	0.59	0.89	0.90	0.75	0.79

Table displaying the mean accuracy and RTs for the 8 words averaged across participants [RT outliers greater than 3000 ms were removed, as was also done in the *C(t)* analyses]. Overall, mean RTs and accuracy were consistent across stimuli within any given condition. Some noticeable exceptions include the mean RTs for “Job” and “Page” relative to the other stimuli in the “A_Med” (−12 dB) condition. One explanation for this particular finding was that the stimuli was that “Job” was often confused with “Shop,” leading to lower mean AV and A accuracy (in the −12 and −18 dB conditions) and longer RTs compared the other stimuli. Nonetheless, the same trend of faster audiovisual vs. auditory or visual-only RTs was observed for all stimuli (including “Job”), in the −12 and −18 dB conditions. These mean RT and accuracy data add converging evidence to the capacity and “Gain” data, which indicate considerable audiovisual benefit in lower S/N ratios, and absence of benefit in optimal listening conditions.
